# Nicotine Suppresses the Invasiveness of Human Trophoblasts by Downregulation of CXCL12 Expression through the Alpha-7 Subunit of the Nicotinic Acetylcholine Receptor

**DOI:** 10.1007/s43032-019-00095-4

**Published:** 2020-01-13

**Authors:** Jing Chen, Min Qiu, Zirui Huang, Jimei Chen, Chengbin Zhou, Fengzhen Han, Yanji Qu, Sheng Wang, Jian Zhuang, Xiaohong Li

**Affiliations:** 1 Guangdong Cardiovascular Institute, Guangdong Provincial Key Laboratory of South China Structural Heart Disease, Guangdong Provincial People’s Hospital, Guangdong Academy of Medical Sciences,School of Medicine, South China University of Technology, Guangzhou, 510080 China; 2 Research Department of Medical Science, Guangdong Provincial People’s Hospital, Guangzhou, China; 3 Department of Cardiovascular Surgery, Guangdong Cardiovascular Institute, Guangdong Provincial People’s Hospital, Guangdong Academy of Medical Sciences, Guangzhou, 510080 China; 4 Department of Obstetrics and Gynecology, Guangdong Provincial People’s Hospital, Guangdong Academy of Medical Sciences, Guangzhou, 510080 China; 5 Department of Epidemiology, Guangdong Cardiovascular Institute, Guangdong Provincial People’s Hospital, Guangdong Academy of Medical Sciences, Guangzhou, 510080 China; 6 Department of Anesthesiology, Guangdong Cardiovascular Institute, Guangdong Provincial People’s Hospital, Guangdong Academy of Medical Sciences, Guangzhou, 510080 China

**Keywords:** Nicotine, Invasion, nAChR, CXCL12, Trophoblast

## Abstract

Smoke exposure during pregnancy has detrimental effects upon numerous fetal and neonatal outcomes. Nicotine (the main component of tobacco) has been suggested to affect placental development. During placental development, efficient invasion by trophoblasts is required for establishment of the fetus–maternal circulation. In this study we explored the regulation of trophoblast invasion by nicotine. An immortalized first trimester extravillous trophoblast cell line (HTR-8/SVneo cells) was used for all the experiments, which were treated by nicotine, methyllycaconitine, and C-X-C motif chemokine ligand 12 (CXCL12). Total RNA and protein were used to study the expressions of nicotinic acetylcholine receptors (nAChRs), and transwell assay was used to study invasiveness. Changes of RNA expression due to nicotine treatment were detected by RNA sequence. Level of CXCL12 mRNA was verified by quantitative PCR. We showed that HTR-8/SVneo expressed subunits α2–4, α7, α9, β1, and β2 of nAChRs. Nicotine downregulated CXCL12 expression and inhibited trophoblast invasion. Methyllycaconitine, as an antagonist of the α7 homopolymer, blocked the inhibitory effect of nicotine. CXCL12 could rescue the nicotine-induced inhibitory effect on invasion of HTR-8/SVneo cells. These results suggest that the α7 subunit of the nAChR has important roles in modulating trophoblast invasion through CXCL12.

**Electronic supplementary material:**

The online version of this article (10.1007/s43032-019-00095-4) contains supplementary material, which is available to authorized users.

## Introduction

Maternal exposure to environmental tobacco smoke (ETS) has been shown to be an important risk factor for pregnancy complications in several epidemiology studies [1, 2]. The negative effects of ETS during pregnancy are abruptio placenta, placenta previa, and intrauterine growth restriction, as well as an increased risk of heart, breathing, and brain abnormalities in the fetus [3–5].

A reduction in the diameter of chorionic villi within the placenta, abnormal apoptosis of trophoblast cells, calcification, DNA methylation, and arterial resistance in the umbilical cord can occur with ETS exposure [6–10]. As an alkaloid found in cigarette smoke, nicotine has been hypothesized to lead to the final downstream histologic changes stated above. The nicotine concentration that perfuses through the placenta is 15% higher in the fetal circulation than that in the maternal circulation [11]. Nicotine binds to nicotinic acetylcholine receptors (nAChRs). nAChRs belong to the cys-loop family of ligand-gated ion channels and include 16 subunits (α1–10, β1–4, δ, ε, and γ) in mammals [12].

nAChRs are expressed predominantly throughout the nervous system [13]. Several studies have measured expression of nAChRs in the human placenta, where α1–7, α9, α10, β1–4, δ, ε, and γ subunits have been reported [14, 15]. However, few studies have focused on nAChRs in trophoblasts.

The invasiveness of trophoblasts is related closely to vascular remodeling in the placenta. Insufficient invasion of trophoblasts can result in an abnormal blood supply to the fetus, thereby resulting in fetal birth defects [16]. However, the effect of nicotine on trophoblasts has not been deeply studied. C-X-C motif chemokine ligand 12 (CXCL12) is an important chemokine on regulating trophoblast phenotypes during the first trimester [17–19]. Nonetheless, whether nicotine can influence trophoblast through regulating CXCL12 expression is unknown.

We wished to define changes in the proliferation and invasion of trophoblast that may occur upon nicotine exposure. Analyses of placental alterations induced by nicotine experimentally could enable characterization of the mechanisms involved in the development of human placental abnormalities under ETS.

## Materials and Methods

### Cell Culture

The trophoblast line HTR-8/SVneo cells (ATCC® CRL-3271™, VA, USA) were cultured in an atmosphere of 5% CO_2_/95% air in Roswell Park Memorial Institute (RPMI) 1640 medium using 75-cm^2^ cell culture flasks (430720; Corning, Corning, NY, USA) and six-well plates. RPMI 1640 medium was supplemented with 5% fetal bovine serum, and the medium was changed every 3 days. HTR-8/SVneo cells were treated with 0.1, 1, or 10-μM nicotine respectively. Methyllycaconitine (MLA, ab120027, Abcam, Cambridge, MA) is an antagonist of the α7 subunit of the nAChR [20] and was used at 10 μM to inhibit nicotine function. C-X-C motif chemokine 12 (CXCL12; catalog number HY-P7287, MedChemExpress, Princeton, NJ, USA) at 100 ng/mL was used to rescue the inhibitory effect of nicotine in HTR-8/SVneo cells.

### Total RNA Extraction and Quantitative Real-Time Polymerase Chain Reaction Analysis

Total RNAs were extracted and purified using TRIzol® Reagent according to manufacturer’s (Invitrogen, Carlsbad, CA, USA) instructions and reverse-transcribed using an Omniscript™ Reverse Transcriptase kit (Qiagen, Valencia, CA, USA) with oligo dT primers. “Hot start” RT-PCR was carried out using a Taq PCR Master Mix kit according to manufacturer’s (Qiagen) instructions. RT-PCR was carried out on a MyiQ™ Single-Color Real-Time PCR detection system (Bio-Rad Laboratories, Hercules, CA, USA) with SYBR® Green Supermix (Bio-Rad Laboratories). Relative levels of expression in each assay were obtained by normalizing the Ct values of the tested genes against that of Tubulin. The primer sequences used for mRNA expression are listed in Supplementary Table 1.

### Western Blotting

Total proteins were extracted from HTR-8/SVneo cells using radioimmunoprecipitation assay (RIPA) lysis buffer. Protein lysates (30 μg) were electrophoresed on 5–10% sodium dodecyl sulfate-polyacrylamide gels and then electroblotted onto polyvinylidene fluoride (PVDF) membranes (Millipore, Waltham, MA, USA). PVDF membranes were blocked with 5% milk in Tris-buffered saline with Tween (TBST) for 1 h and incubated with primary antibodies against nAChRs overnight at 4 °C (Abcam, Cambridge, UK). Then, PVDF membranes were washed by TBST and incubated with secondary antibodies for 45 min at 4 °C. After washing, membranes were incubated with enhanced chemiluminescence (ECL) detection solution (Cat. 34080, Supersignal West Pico Chemiluminescent Substrate, Thermo, USA) and exposed to X-ray film. The details of the primary and secondary antibodies were showed in Supplementary Table 2.

### Immunofluorescence Microscopy

Cells were washed with phosphate-buffered saline (PBS), fixed in 4% paraformaldehyde overnight and permeabilized with 0.1% Triton X-100/PBS. Slides were blocked for 1 h in blocking solution and incubated overnight at 4 °C with primary antibodies against nAChRs. IgG-isotype control was used as negative control. After washing in PBS, slides were incubated with secondary antibodies. Nuclear counterstaining was done with 4′,6-diamidino-2-phenylindole. Slides were washed, mounted, and viewed through a laser scanning confocal microscope (SP5-FCS; Leica, Wetzlar, Germany). The details of the primary and secondary antibodies were also showed in Supplementary Table 2.

### Cell-Viability Assay

HTR-8/SVneo cells (5 × 10^3^ cells/well) were seeded into 96-well plates. Cell viability was analyzed by the 3-(4,5-dimethylthiazol-2-yl)-2,5-diphenyltetrazolium (MTT) assay after nicotine exposure. In brief, HTR-8/SVneo cells were treated with nicotine (0.1, 1, or 10 μM) added to the culture medium. At the end of culture at 24 h, 48 h, and 72 h, 50 mL of MTT solution (0.5 mg/mL) was added to the medium and incubation allowed to proceed for 4 h at 37 °C. Then, the medium was replaced with 150 mL of dimethyl sulfoxide to dissolve formazan crystals in the absence of light but with agitation for 15 min. The MTT reaction was determined by spectroscopy at 570 nm on a microplate reader (Multiskan™ GO, Thermo Scientific, Waltham, USA). The experiment was repeated four times.

### Transwell™ Cell-Invasion Assay

A serum-free suspension of cultured cells was loaded onto the Matrigel™-coated upper parts of Transwells (pore size of 8 μm; 5 × 10^4^cells/insert; Costar; Corning) in triplicate. The lower parts of the Transwells contained culture media containing 10% fetal bovine serum. The assembled 24-well plates were incubated for 18 h in a humidified atmosphere of 5% CO_2_. Invading HTR-8/SVneo cells that had entered the lower surface of the filter membrane were stained with Wright-Giemsa Stain Kit (Nanjing JianCheng Technology co., LTD, D010-1-2, China), photographed using a light microscope (Eclipse; Nikon; Tokyo, Japan), and counted by using ImageJ (San Diego, CA, USA), whereas non-invading HTR-8/SVneo cells were removed carefully with a cotton swab. Each assay was carried out in triplicate wells.

### Construction of an RNA Library and RNA Sequencing

RNA sequencing libraries were prepared using an Epi™ RNA Library Fast kit (Epibiotek, Beijing, China). Briefly, RNA was fragmented using Mg^2+^ and first- and second-strand cDNA synthesis allowed to proceed using random hexamer primers. cDNA fragments were passed onto end-repair, dA-tailing, and adapter-ligation steps. After size selection of adaptor-ligated DNA, purified dsDNA was subjected to 15 cycles of PCR amplification. The DEGseq algorithm was applied to filter differentially expressed genes under the criteria of log2 FC (fold change) > 1 and FDR (false discovery rate) < 0.05. FDR was used to correct *p* values. Volcano plots were drawn by the R program (R Computing, Vienna, Austria) based on differentially expressed genes, and the color was determined using the filtering criteria. Functional enrichment using Gene Ontology (GO) and Kyoto Encyclopedia of Genes and Genomes (KEGG) databases was analyzed for mRNAs in the predictive signature to reveal the potential functions of nicotine. Fisher’s exact test was applied to identify the significant categories and pathways according to the GO database.

### Statistical Analyses

Statistical analyses were undertaken using Prism 7 (GraphPad, San Diego, CA, USA). Quantitative data were analyzed using the two-tailed Student’s *t* test for determination of differences in mean values between two groups and one-way analysis of variance (ANOVA) followed by Tukey’s honestly significant difference test. Results are the mean ± SD. *p* < 0.05 was considered significant.

## Results

### Expression of nAChR Subunits in HTR-8/SVneo Cells

**Fig. 1:**
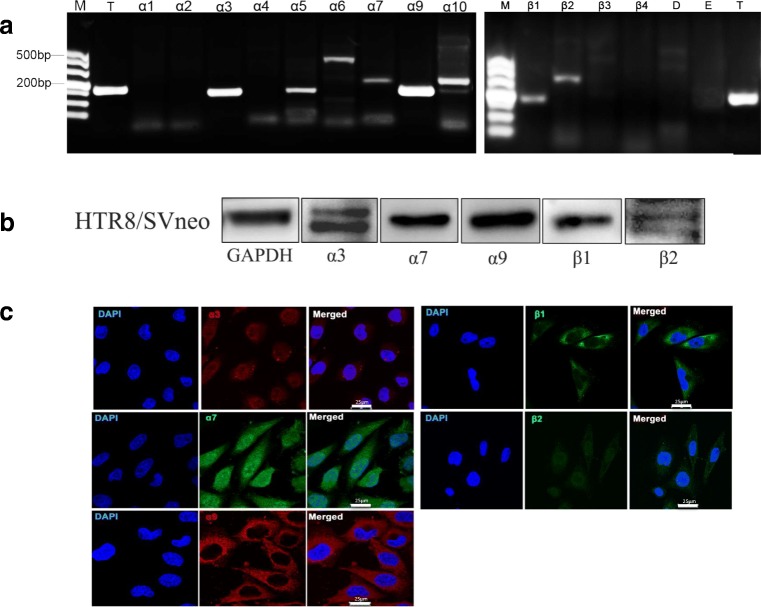
Expression of nAChRs in HTR-8/SVneo cells. **a** The expression of nAChR subunits specific mRNA in HTR-8/SVneo cells was detected by RT-PCR. α1–α7, α9, and α10 were α subunits of nAChR. β1–β4 were β subunits of nAChR. D Subunit. E Subunit. T Tubulin was used as internal control. M 500 bp DNA marker. **b** Expression of nAChRs protein was detected by Western blotting. GAPDH was used as internal control. **c** Representative fluorescent images were shown with nAChRs in HTR-8/SVneo cells. 4′,6-diamidino-2-phenylindole (DAPI_ staining was performed to visualize nuclei (blue). Scale bar, 25 μm. Magnification, × 200

At the mRNA level, the nAChR subunits α3, α5, α6, α7, α9, α10, β1, and β2 were expressed in HTR-8/SVneo cells. Most bands were detected clearly at the correct size by RT-PCR (Fig. 1a). Specific bands of α3, α7, α9, and β1 subunits were observed at the protein level by western blotting. However, the β2 band was dim and smeared (Fig. 1b). Bands for α5, α6, or, α10 subunits were not detected. Immunofluorescence staining displayed expression of α3, α7, α9, β1, and β2 subunits in HTR-8/SVneo cells. However, the α3 subunit was located primarily in the nucleus. The fluorescence intensity of the β2 subunit was very weak. The α9 and β1 subunits were located in the cytoplasm. The α7 subunit was located in the nucleus and cytoplasm (Fig. 1c). IgG was used as isotype control (SFig. 1). The α7 subunit of the nAChR has an important regulatory role in non-neural cells [21, 22]. So, subsequent studies focused on the function of α7 homopolymer of the nAChR.

### Cell Viability Is Not Affected by Nicotine

**Fig. 2:**
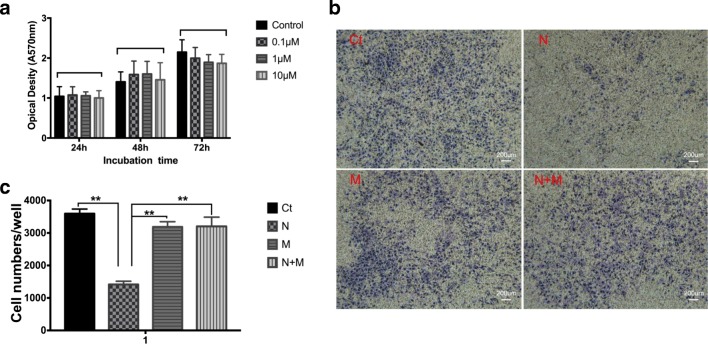
The effects of nicotine on HTR-8/SVneo cell viability and invasion. **a** MTT assays were performed for cell viability after nicotine treatment at different time. Nicotine concentration: 0.1 μM, 1 μM, and 10 μM. Data represent the means of four independent experiments. Error bars are standard deviations. **b** Cell invasion detected by Transwell assay at 18h. Ct, control; N, 1-μM nicotine; M, methyllycaconitine (MLA); N+M, nicotine + MLA. Magnification × 50. Scale bar 25 μm. **c** Counting of violet cells in the whole well by fluorescent microscopy using the ImageJ software. Data were shown as mean ± SD. **p < 0.01 vs nicotine group

The viability of HTR-8/SVneo cells treated with nicotine (0.1, 1, 10 μM) was measured by the MTT assay. Compared with the untreated group, the MTT activity in HTR-8/SVneo cells exposed to nicotine (0.1, 1, 10 μM) at 72 h was increased slightly (Fig. 2a) but not significantly (*p* > 0.05). Nicotine at 1 μM is close to the physiologic concentration of nicotine in plasma in people exposed to tobacco. This finding suggested that 1 μM of nicotine did not interfere with the survival of HTR-8/SVneo cells and, thus, could be used to study its physiologic relevance in nAChRs.

### Invasiveness of HTR-8/SVneo Cells Was Inhibited by Nicotine

We investigated the effect of nicotine on the invasiveness of HTR-8/SVneo cells using Transwells. Nicotine inhibited the ability of HTR-8/SVneo cells to invade, but this ability could be rescued by MLA (Fig. 2b). The number of HTR-8/SVneo cells invading spontaneously in Transwells was decreased dramatically in nicotine-supplemented cultures, but restored upon MLA treatment (*p* < 0.01) (Fig. 2c). This result suggested that nicotine suppressed the invasiveness of HTR-8/SVneo cells. These effects of nicotine upon the invasiveness of HTR-8/SVneo cells appeared to be mediated by the α7 subunit of the nAChR because the inhibitory effects were blocked by MLA.

### RNA Sequencing of HTR-8/SVneo Cells after Nicotine Treatment

**Fig. 3:**
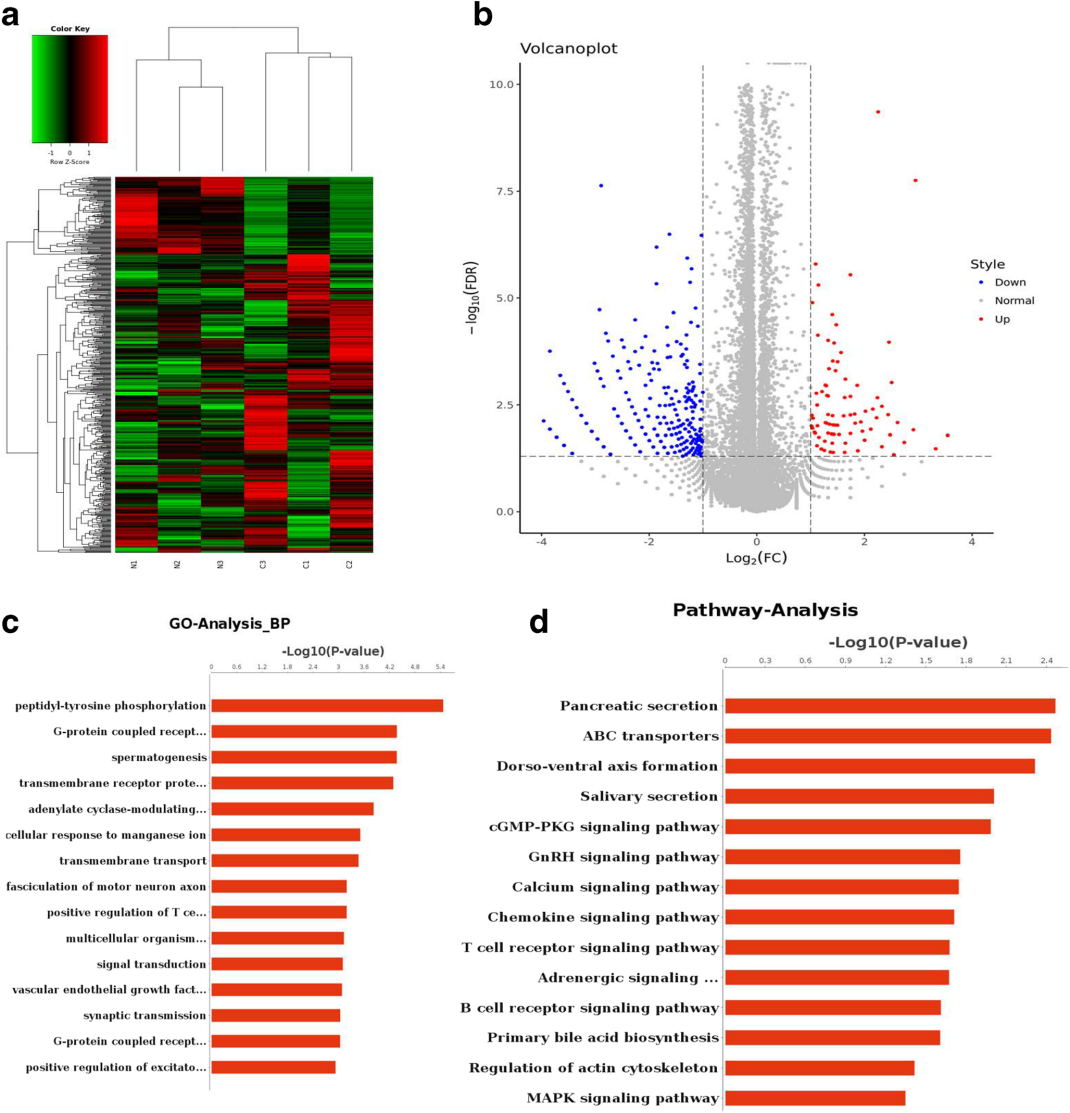
Difference of RNA expression detected by sequencing between control and nicotine group. **a** Heat map of RNA sequencing profiles of HTR-8/SVneo cells from control and nicotine groups. N1, N2, N3, 1-μM nicotine groups. C1, C2, C3, control groups. Red upregulated genes. Green down-regulated genes. Black normal expressed genes. **b** Differentially expressed genes displayed by Volcano Plots. Red spots upregulated genes. Blue spots downregulated genes. Gray spots normally expressed genes. **c** Gene ontology (GO) analysis was performed to facilitate elucidating the significantly changed biological process (BP) unique genes (*p* < 0.01). **d** Significant pathways of the differential genes according KEGG pathway analysis (*p* < 0.01)

RNA sequencing was conducted to identify mRNAs that might be related to the phenotypic changes during invasion by HTR-8/SVneo cells under nicotine treatment. The major annotated genes were clustered by hierarchical clustering analysis (Fig. 3a). Expression of 415 genes was downregulated dramatically and expression of 108 genes was upregulated dramatically after nicotine treatment (Fig. 3b). These results suggested that the altered expression of these genes was enriched in 272 biologic process (BP) terms in the GO database. These were clustered mainly in regulation of diverse BPs in the GO database (e.g., 0007186: regulation of a G protein-coupled receptor signaling pathway; 0007165: signal transduction; 0007169: transmembrane receptor protein tyrosine kinase) (Fig. 3c). Fourteen downregulated pathways in the KEGG database were enriched (e.g., PATH: 02010 ATP-binding cassette (ABC) transporters; PATH: 04062 chemokine signaling pathway) (Fig. 3d). The modulated genes were listed in Supplementary Table 3.

### Changes in CXCL12 Expression Are Related to the Invasiveness of HTR-8/SVneo Cells under Nicotine Treatment

**Fig. 4:**
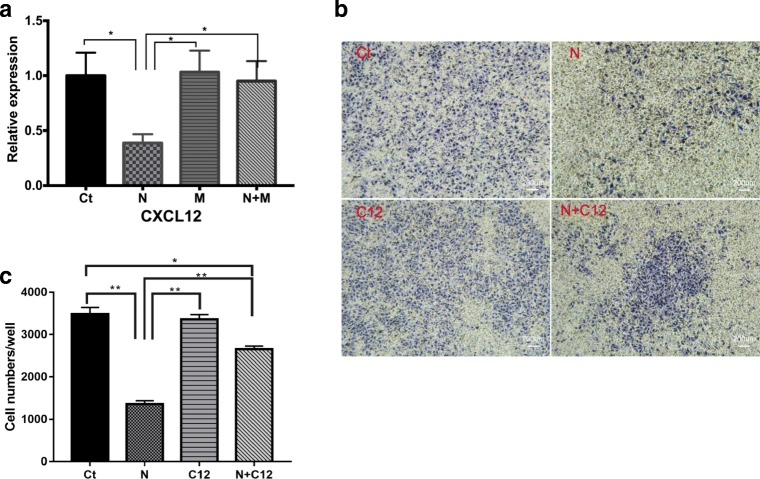
Subunit α7 is associated with CXCL12 expression and cell invasion. **a** CXCL12 is detected by qPCR after treatment with 1-μM nicotine and/or MLA for 12 h. Error bars are standard deviations. GAPDH was used as internal control. Ct Control. N 1-μM nicotine. M MLA. N+M Nicotine + MLA. *p < 0.05 vs nicotine group. **b** Cell invasion was detected after CXCL12/nicotine treatment at 18 h. **a** Ct Control; N 1 μM nicotine; C12 CXCL12; N+C12 nicotine + CXCL12. Magnification × 50. Scale bar 200 μm. **c** Counting of violet cells in the whole field by fluorescent microscopy using the ImageJ software. Data were shown as mean ± SD. *p < 0.05 vs control; **p < 0.01 vs nicotine group

*CXCL12* was involved in a G protein-coupled receptor signaling pathway and chemokine signaling pathway (Supplementary Table 3). CXCL12 expression was decreased according to RNA-sequencing data, and this result was confirmed further by RT-qPCR (Fig. 4a). Expression of CXCL12 mRNA was downregulated significantly after nicotine treatment as compared with that in the control group (*p* < 0.05). MLA did not affect CXCL12 expression. If combined with nicotine, MLA significantly rescued the reduced CXCL12 expression (*p* < 0.05).

### Invasion by HTR-8/SVneo Cells After CXCL12 Treatment

To explore the functional role of CXCL12 in HTR-8/SVneo cells, we conducted assays to assess the invasiveness of HTR-8/SVneo cells using recombinant human CXCL12. In each assay, HTR-8/SVneo cells placed on the upper parts of Transwell inserts were treated with 100 ng/mL of CXCL12, followed by quantification of invasion after incubation for 18 h. Invasion by HTR-8/SVneo cells was increased significantly after CXCL12 treatment in the nicotine group, while the group treated by CXCL12 alone had no significance with the control group (Fig. 4b). CXCL12 increased the number of migrated HTR-8/SVneo cells when compared with that in the nicotine group (*p* < 0.01). However, CXCL12 restored only partially the number of invading HTR-8/SVneo cells compared with that in the control group (*p* < 0.01) (Fig. 4c).

## Discussion

Expressions of α1–7, α9, α10, β1–4, δ, ε, and γ subunits of nAChR in normal healthy placenta tissue have been reported before [14]. In the present study, analyses of reverse-transcribed mRNA for all nAChR subunits tested resulted in expression of α3, α5, α6, α7, α9, α10, β1, and β2 subunits, but not of α1, α2, α4, β3, β4, δ, or ε subunits, in human HTR-8/SVneo cells. Protein expression of α3, α7, α9, β1, and β2 subunits was shown by western blotting and immunofluorescence data. The immunofluorescence pictures showed a different distribution of these nAChR subunits. The α3 subunit was primarily found in the nucleus; the α7 subunit was located in the cytoplasm and nucleus, but α9 and β1 subunits are expressed in the cytoplasm in this study.

The α7 and α9 subunits usually form homopentameric receptors, whereas other subunits combine into heteropentameric structures with α and β subunits [13]. We detected only low expression of β1 and β2 subunits. Expression of β3 or β4 subunits at the protein level was not observed. These data may suggest a low component of the heteropentameric structure of nAChRs in HTR-8/SVneo cells. Expression of the α7 subunit revealed in our study was consistent with that reported previously in the human placenta [23–25]. The α7 subunit has been identified biochemically in the human placenta, and its central role in mediating cell motility was investigated by Schraufstatter and colleagues [22]. However, involvement of the α7 subunit with specific cellular processes has not been defined. Our study provides data on the importance of the α7 subunit in human trophoblasts.

At 0.1 to 10 μM, nicotine did not influence trophoblast proliferation. We know that 1 μM is close to the mean physiologic concentration of nicotine measured in the blood of tobacco smokers [26], so this concentration was used to test the physiologic effects of nicotine on trophoblast function. Schraufstatter and colleagues demonstrated that nicotine inhibits the motility of mesenchymal stem cells [22], so we tested the role of nAChRs in regulating invasion by HTR-8/SVneo cells. nAChRs were stimulated with 1 μM of nicotine because we found that this concentration did not influence the survival of HTR-8/SVneo cells significantly.

There have been contradictory reports regarding the effects of nicotine upon cell motility. Some research teams have reported that nicotine stimulates the migration or invasion of cells [27–29], whereas other scholars have demonstrated that nicotine is an inhibitory factor [22, 30]. Nicotine induces signal transduction in non-neural cells, which can be associated with increased cell motility. However, the signaling mechanisms activated might have been dependent upon the specific cell types and diverse experimental conditions used in those studies. We found that spontaneous invasion of HTR-8/SVneo cells across the membrane was inhibited significantly if 1 μM of nicotine was added to cultures. The inhibitory effect of nicotine upon invasion by trophoblasts in vitro was attenuated in the presence of MLA, which suggested a role for the α7 subunit of the nAChR in mediating these effects of nicotine.

CXCL12 is an important chemokine for cell motility [31]. Zhang and colleagues showed that nicotine can inhibit CXCL12 expression [32]. We showed that *CXCL12* expression was downregulated by nicotine in HTR-8/SVneo cells. Primary trophoblasts secrete high levels of CXCL12 to promote their own invasiveness and matrix-metalloproteinase activity [33, 34]. Several studies have reported that CXCL12 is crucial for regulating trophoblast phenotypes during the first trimester [18, 35]. Our study suggested that inhibition of *CXCL12* expression by nicotine is a novel regulatory system that could influence the invasiveness of HTR-8/SVneo cells. This regulatory system may be important for extravillous trophoblast invasion into the maternal decidua, myometrium, or spiral arteries, and impaired invasion could cause inadequate vascular remodeling and subsequent poor placentation. However, CXCL12 restored the number of invasive HTR-8/SVneo cells only partially compared with that in the control group. These data suggest there may be other mechanisms by which nicotine inhibits trophoblast invasion.

Our study had two main limitations. Firstly, we could not elucidate the precise mechanism underlying CXCL12 inhibition after nicotine treatment in this trophoblast cell line. Secondly, it is possible that nicotine inhibits invasiveness of HTR-8/SVneo cells and expression levels of CXCL12 through other subunits of nAChR, and it needs further investigation. Lastly, all experiments were conducted in only one cell line, so additional studies are needed to determine the physiologic and pathologic roles of the α7 subunit of the nAChR and CXCL12 during placentation in vivo.

In future studies, we wish to ascertain the potential of CXCL12 as a therapeutic target/diagnostic marker for the pregnancy complications related to tobacco smoking. In conclusion, our findings suggest that nicotine suppresses the invasiveness of HTR-8/SVneo cells by downregulating CXCL12 expression through the α7 subunit of the nAChR. We propose that the α7 subunit of the nAChR and CXCL12 have important roles in modulating trophoblast invasion during cigarette smoking.

## Electronic supplementary material

ESM 1 (DOC 470 kb)

ESM 2 (DOC 62 kb)

ESM 3 (XLS 98 kb)

## Abbreviations

CXCL12: C-X-C motif chemokine ligand 12
nAChR: Nicotinic acetylcholine receptor
ETS: Environmental tobacco smoke
GO: Gene ontology
BP: Biologic process
KEGG: Kyoto Encyclopedia of Genes and Genomes
