# Supplementary material for: Nicotine Suppresses the Invasiveness of Human Trophoblasts by Downregulation of CXCL12 Expression through the Alpha-7 Subunit of the Nicotinic Acetylcholine Receptor
Source: Reprod Sci. 2020 Jan 13;27(3):916–24. doi: 10.1007/s43032-019-00095-4 (PMC7077928; doi:10.1007/s43032-019-00095-4)
Supplement: Supplementary file 1 — (DOC 470 kb) [file 43032_2019_95_MOESM1_ESM.doc]

**Supplementary Figure 1**


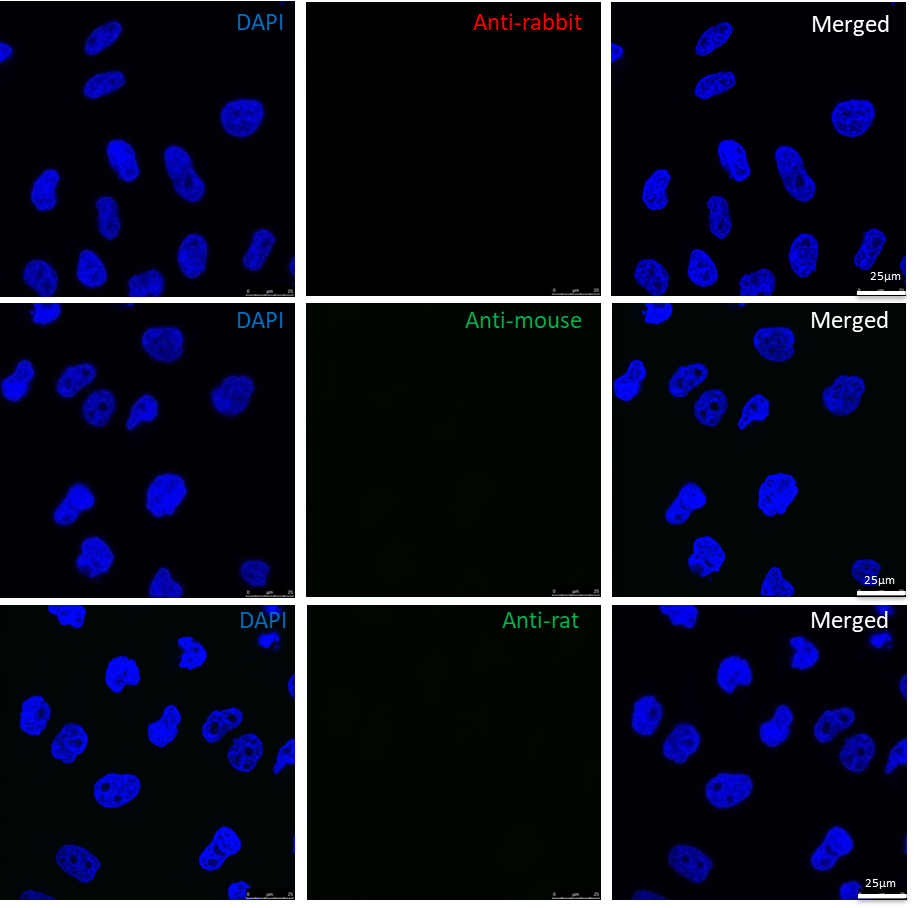


SFig. 1. Negative control of the immunofluorescence stain. Anti-rabbit: rabbit IgG, polyclonal-isotype Control; Anti-mouse: mouse IgG-Isotype Control; Anti-rat: rat IgG-Isotype Control. DAPI was used for nucleic acid staining. (Scale bar: 25μm)
