# Supplementary material for: Nicotine Suppresses the Invasiveness of Human Trophoblasts by Downregulation of CXCL12 Expression through the Alpha-7 Subunit of the Nicotinic Acetylcholine Receptor
Source: Reprod Sci. 2020 Jan 13;27(3):916–24. doi: 10.1007/s43032-019-00095-4 (PMC7077928; doi:10.1007/s43032-019-00095-4)
Supplement: Supplementary file 2 — (DOC 62 kb) [file 43032_2019_95_MOESM2_ESM.doc]

**Supplementary tables**

**Table 1. The primers used for PCR are shown where “F” refers to the forward and “R” to the reverse primers**

| **Genes** | **Primer sequence** | **Product size** |
| --- | --- | --- |
| α1 | F: 5’-CGACACTATCCCAAATATCATG-3’  R: 5’-TTGTTAGACTCCTGGTCTGTC-3’ | 221bp |
| α2 | | F: 5’-CTACGCCTGAAGCTCAGCC-3’ | | --- | | R: 5’-CCTTCACCGAAGAGTCAGC-3’ | | 296bp |
| α3 | F: 5’-CAGAGTCCAAAGGCTGCAAG-3‘  R: 5’-AGAGAGGGACAGCACAGCAT-3 | 149bp |
| α4 | F: 5’-GACTTCGCGGTCACCCACCT-3’  R: 5’-CGGCTGTGCATGTTCACCAG-3’ | 197bp |
| α5 | F: 5’-CTTCACACGCTTCCCAAACT-3’  R: 5’-CTTCAACAACCTCACGGACA-3’ | 187bp |
| α6 | F: 5’-AACCAATTTGTGGCTGCGTC-3’  R: 5’-TGTTTGTAGCCAGAGGCATCA-3’ | 405bp |
| α7 | | F: 5’-AGGGGACAGATGGACTCTGAA-3’ | | --- | | R: 5’-GCCATCTGGGAAACGAACAG-3’ | | 276bp |
| α9 | F: 5’- GAAAGCAGCCAGGAACAAAG-3’  R: 5’-GCACTTGGCGATGTACTCAA-3’ | 157bp |
| α10 | F: 5’-TGCTGGGACACCTGGCACG-3’  R: 5’-TCATGGCAGCGCTGGGCAG-3’ | 234bp |
| β1 | F: 5’-GAGCCCCCTCACTGTTCTTC-3’  R: 5’-ATAGAGGAGACGACCTCCCG-3’ | 206bp |
| β2 | F: 5’- TGCCATCTACAAGAGCGCAT-3’  R: 5’-GCAGCACTGAGATGCACAAC-3’ | 377bp |
| β3 | | F: 5’-CTTTGCATGAAAGATCATGTGG-3’ | | --- | | R: 5’-TGTTCTTTCTTCACATGTCTCG-3’ | | 191bp |
| β4 | F: 5’-TCTTCATGAAGCGCCCTGGC-3’  R: 5’-TCTCTGGTCTTCATCGTCATTC-3’ | 306bp |
| δ | F: 5’-ATGTGTGGATAGAGCACGGC-3’  R: 5’-CTGGAAGGAGCCGTCATTGT-3’ | 146bp |
| ε | F: 5’-TCAGGCCCACAGGTTTAAGG-3’  R: 5’-AATTCAACCAGCTCCCACCAT-3’ | 225bp |
| Tubulin | F: 5’-GCATTCCAACCTTCCAGCCT-3’  R: 5’-ATCACCTCCCAGAACTTGGC-3’ | 171bp |
| GAPDH | F: 5’-TTCTTTTGCGTCGCCAGCC-3’  R: 5’-GACGGTGCCATGGAATTTGCC-3’ | 220bp |
| CXCL12 | F: 5’-TGAAAGGGCAAAGACGTGGG-3’  R: 5’-TCCAACGTGCACAGGTACAG-3’ | 180bp |

**Table 2. The primary and secondary antibodies used in the Western blot/IF.**

| **Antibodies** | **dilution** | **description** |
| --- | --- | --- |
| α3 | WB: 1:1000;  IF: 1:200 | ab151580, Abcam, Cambridge, MA, USA |
| α7 | WB: 1:1000;  IF: 1:200 | MA5-31691, Thermo Fisher, Waltham, MA, USA |
| α9 | WB: 1:1000;  IF: 1:200 | PA5-77511, Thermo Fisher, Waltham, MA, USA |
| β1 | WB: 1:1000;  IF: 1:200 | sc-65790, Santa Cruz, California, USA |
| β2 | WB: 1:1000;  IF: 1:200 | sc-58596, Santa Cruz, California, USA |
| GAPDH | WB: 1:8000 | KC-5G5, Kang Chen, Shanghai, China |
| anti-mouse | WB: 1:8000 | A21010, Abbkine, Wuhan, China |
| anti-rat | WB: 1:8000 | A21020, Abbkine, Wuhan, China |
| anti-rabbit | WB: 1:8000 | A21040, Abbkine, Wuhan, China |
| DAPI | IF: 1:1000 | D9542, Sigma, St Louis, Missouri, USA |
| Anti-Mouse IgG (H+L) (Alexa Fluor® 488 Conjugate) | IF: 1:800 | A11001, Thermo Fisher, Waltham, MA, USA |
| Anti-rat IgG (H+L) (Alexa Fluor® 488 Conjugate) | IF: 1:800 | #4416, Cell Signalling Technology, Denver, Colorado, USA |
| Anti-rabbit IgG-PE | IF: 1:400 | sc-3739, Santa Cruz, California, USA |
| Rabbit IgG, polyclonal-isotype Control | IF: 1:200 | Ab37415, Abcam, Cambridge, MA, USA |
| Mouse IgG-Isotype Control | IF: 1:200 | Ab37355, Abcam, Cambridge, MA, USA |
| Rat IgG-Isotype Control | IF: 1:200 | Ab37361, Abcam, Cambridge, MA, USA |
